# PUFA, fish intake and risk of disabling dementia in Japan: the Japan Public Health Centre Disabling Dementia Study

**DOI:** 10.1017/S1368980025000308

**Published:** 2025-03-20

**Authors:** Sarah K Abe, Manami Inoue, Nobufumi Yasuda, Kazumasa Yamagishi, Shoichiro Tsugane, Norie Sawada, for the JPHC Study Group

**Affiliations:** 1 Division of Prevention, National Cancer Center Institute for Cancer Control, Tokyo, Japan; 2 Cancer Epidemiology, The University of Tokyo, Tokyo, Japan; 3 Division of Cohort Research, National Cancer Center Institute for Cancer Control, Tokyo, Japan; 4 Department of Public Health, Kochi University Medical School, Kochi, Japan; 5 Department of Public Health Medicine, Institute of Medicine, and Health Services Research and Development Center, University of Tsukuba, Tsukuba, Japan; 6 Department of Public Health, Graduate School of Medicine, Juntendo University, Tokyo, Japan; 7 International University of Health and Welfare Graduate School of Public Health, Tokyo, Japan

**Keywords:** Fish, PUFA, Dementia, Japan, Cohort

## Abstract

**Objective:**

The aim of this study was to assess the association between fish intake, *n*-3 PUFA, *n*-6 PUFA and risk of disabling dementia.

**Design:**

Prospective cohort.

**Setting:**

Municipalities within the Japan Public Health Centre-based Prospective Study.

**Participants:**

43 651 participants: (20 002 men and 23 649 women).

**Results:**

Exposure intake of fish, *n*-3 and *n*-6 PUFA was evaluated in 1995–1997. We defined disabling dementia cases as participants who were certified to receive disability care under the long-term-care insurance programme (2006–2016) in participating municipalities with a grade of activities of daily living related to dementia ≥ IIa on the dementia rating scale (range 0–IV and M). Cox proportional hazard models were applied to obtain hazard ratios (HR) and 95 % CI according to quartiles of exposures of interest. In the main analysis, we adjusted for age and area, smoking, BMI, alcohol and metabolic equivalent tasks. During 410 350 person-years of follow-up with an average follow-up of 9·4 years, 5278 cases of disabling dementia were diagnosed. Fish intake and most PUFA were not associated with the risk of disabling dementia in men. In women, *n*-6 PUFA showed a significant decreasing trend in risk the highest HR (95 % CI) compared with the lowest was 0·90 (0·81, 0·99) (*P* for trend = 0·024) and alpha-linolenic acid (ALA) was 0·91 (0·82, 1·00) (*P* for trend = 0·043).

**Conclusions:**

Our findings suggest no association with fish in general and only *n*-6 PUFA and ALA may be associated with a decreased risk of disabling dementia especially in women.

Globally, 50 million people are living with dementia^(1)^. This number is likely to triple by 2050, with around 10 million new cases every year^(1)^. The WHO has listed dementia as a public health priority^(1)^. Japanese healthcare policy prioritises dementia prevention, control and care through a comprehensive strategy implemented in 2015^(2)^. Although the Japanese enjoy a high life expectancy, they also experience long years of activity limitation, at 8·4 years for men and 11·8 years for women^(3)^.

Various modifiable risk factors for dementia have been explored, including physical inactivity^(4)^, obesity, unhealthy diet, tobacco, high levels of alcohol consumption^(5)^, low educational attainment, social isolation/depression and cognitive impairment, hearing loss, high blood pressure, high blood glucose and glucose metabolism impairment, among others^(6)^. Associations between dietary factors and dementia are inconsistent. Addressing these modifiable risk factors may allow dementia to be partially prevented, its onset delayed or the severity of progression reduced. Previous epidemiological research on diet-related factors^(7)^ such as fish is inconsistent^(8–10)^. Sex-specific differences should be considered to identify underlying risk mechanisms related uniquely to men or women as dietary patterns and dementia risk may be sex-dependent^(11)^.

Fish may be a modulating risk factor for dementia. Fish consumption in Japan is high but has been decreasing^(12)^. A comprehensive meta-analysis of twenty-one cohort studies, which included over 180 000 participants concluded that one serving per week increment of fish was associated with a 10 % lower risk of dementia^(13)^. These results were echoed by a 2018 systematic review^(14)^ and a Japanese cohort study^(15)^. In contrast, a meta-analysis of forty-three cohort studies published in 2016 found no such association^(16)^. Neither of these reviews included any Japanese studies. Another meta-analysis from 2015 reported no association between fish and *n*-3-fatty acid intake and dementia, although higher fish intake was associated with a 36 % lower risk of Alzheimer’s disease^(17)^. Although Japanese and Korean epidemiological studies additionally suggest that alpha-linolenic acid (ALA), plant-derived *n*-3 PUFA, as well as the fish-derived *n*-3 PUFA DHA, docosapentaenoic acid (DPA) and EPA may be associated with dementia^(10,18,19)^, few studies to date have evaluated fish intake and dementia risk in the Japanese population, whose consumption of fish is relatively high. This association therefore warrants evaluation.

Overall reasons for the inconsistency in the association of fish (and fish-related nutrients) with dementia among previous epidemiological studies may partially include variation in the quantity and type of fish consumed, as well as other factors. This study is unique to all other previous studies in that the data come from a large Japanese cohort with many disabling dementia cases allowing for more refined sex-specific analyses in eight relevant exposures (fish, PUFA-rich fish, *n*-6 PUFA, *n*-3 PUFA, EPA, DHA, DPA and ALA). Participants’ fish consumption was high overall compared with studies conducted in other countries. Reflecting on the variations, although fish is not the major source of *n*-6 PUFA, we included this group of nutrients in this study as in previous studies^(20)^.

Here, we aimed to assess the association between fish, *n*-3 PUFA and *n*-6 PUFA intake and the risk of disabling dementia in a large community-based cohort, The Japan Public Health Centre-based-Prospective study (JPHC study).

## Methods

### Study population

The JPHC study was initiated in 1990 among Japanese residents aged 40–59 years living in Akita, Iwate, Nagano, Okinawa and Tokyo prefectures (Cohort I). Cohort II was added in 1993 among Japanese residents aged 40–69 residing in Ibaraki, Kochi, Nagasaki, Niigata, Okinawa and Osaka prefectures. The two cohorts including 140 420 participants are profiled in detail elsewhere,^(21)^ and the questionnaire surveys were repeated every 5 years.

**Figure 1.**
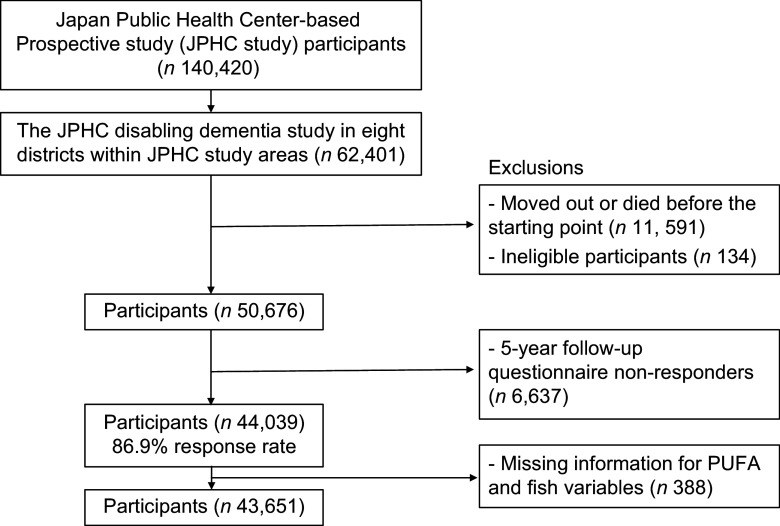
Flowchart of study participants.

The present study included eight areas: the Omonogawa and Yokote areas in Yokote city in Akita prefecture, the Iwase area in Sakuragawa city and Tomobe area in Kasama city in Ibaraki prefecture, the Usuda area in Saku city in Nagano prefecture, the Kagami and Noichi areas in Konan city in Kochi prefecture and the Gushikawa area in Uruma city in Okinawa prefecture. We excluded non-Japanese participants (fifty-one participants), late reports of migration (*n* 65), incorrect birthdate (*n* 3), duplicate registration (*n* 4), refusal of follow-up (*n* 11) and those who moved out or died before the starting point of the dementia ascertainment period, 2006 (11 591 participants) with the exception of Saku city (follow-up for dementia commenced in 2009), leaving 50 676 participants (Figure 1). Of these, 86·9 % responded to the questionnaires administered from 1995 to 1997. Participants with missing fish and fish-related nutrient exposure data were excluded (*n* 388). Finally, 43 651 (20 002 men and 23 649 women) were included in this analysis. The JPHC study did not obtain individual consent but provided opt-out opportunity.

### Disabling dementia case ascertainment

The present study utilised certification records of the long-term care insurance system to identify study participants with disabling dementia, details published elsewhere^(4,5,7)^. Incident dates were defined by the initial certification date. Long-term care insurance certification records for this study were available for the period between January 1, 2006 and December 31, 2016, with the exception of Saku City starting in 2009. The Ministry of Health, Labor and Welfare of Japan introduced the insurance system in 2000, and it is administered by municipalities^(22)^. Residents aged 65 and older and those with disability aged 40–64 wishing to use long-term care services must undergo certification as functionally disabled by application to their municipality for support/long-term care. The municipality assesses the applicant’s functional health status via a comprehensive assessment and obtains a primary care physician’s written opinion about the applicant’s disability. We defined disabling dementia as certification at a level indicating long-term care (levels 1–5) and within the range of severity of cognitive disability (grade IIa, IIb, IIIa, IIIb, IV or M) on the dementia rating scale as derived from the primary care physician’s written opinion.

### Exposure assessment

We used the self-administered 5-year follow-up survey FFQ of the JPHC study in 1995–1997. The survey included questions about the frequency and portion size of 138 food and beverage items^(23)^. We included nineteen seafood item-related questions from the questionnaire: salted fish, dried fish, canned tuna, salmon or trout, bonito or tuna, cod or flat fish, sea bream, horse mackerel or sardine, mackerel pike or mackerel, dried small fish, eel, salted roe, prawn, squid, octopus, short-necked clam or crab shell, vivipara, kamaboko (fish paste product) and chikuwa (fish paste product). Of these, eleven were considered fish (salted fish, dried fish, canned tuna, salmon or trout, bonito or tuna, cod or flat fish, sea bream, horse mackerel or sardine, mackerel pike or mackerel, dried small fish and eel). Five of these eleven were categorised as PUFA-rich fish (horse mackerel or sardine, mackerel pike or mackerel, sea bream, salmon or trout and eel)^(24)^. Participants documented their average frequency and portion size from the year prior to the survey by choosing from the following categories (never, 1–3 times/month, 1–2 times/week, 3–4 times/week, 5–6 times/week, once/day, 2–3 times/day, 4–6 times/day and 7 or more times/day)^(25)^. Standard portions were considered small (50 % smaller), medium (same as standard) and large (50 % larger). Food intake (grams/day) was calculated by multiplying frequency by standard portion size. FFQ information using all 138 food items was converted into nutrient intake to calculate the daily intake of *n*-3 PUFA, *n*-6 PUFA, EPA, DHA, DPA and ALA in grams per day. We used a fatty acid composition table based on the respective supplemental Standard Tables of Food Composition in Japan (Fifth revised edition)^(24)^. A subsample of JPHC Study FFQ responses was used to assess validity in 215 subjects with both FFQ and complete 28-day dietary records (DR); 7-day DR four times to account for each season and twice in Okinawa which is subtropical^(25,26)^. Spearman rank correlation coefficients between the FFQ and DR, collected the same years as the FFQ, were 0·21 and 0·34 for total *n*-3, 0·30 and 0·21 for total *n*-6, 0·62 and 0·55 for EPA, 0·32 and 0·39 for DPA, 0·61 and 0·50 for DHA and 0·27 and 0·25 for ALA in men and women, respectively^(26)^. The validity of the FFQ was deemed sufficient^(26)^.

### Statistical analysis

Cox proportional hazard models were used to estimate hazard ratios (HR) and 95 % CI. Participants were divided into sex-specific quartiles for energy-adjusted fish, *n*-3 PUFA (including EPA, DHA, DPA and ALA) and *n*-6 PUFA intake in g/day. Quartiles were selected to determine trends. We calculated the p-values for the interaction between the studied factors and disabling dementia across different genders: fish *P* = 0·061, PUFA-rich fish *P* = 0·101, *n*-3 PUFA *P* = 0·866, *n*-6 PUFA *P* = 0·359, EPA *P* = 0·029, DHA *P* = 0·088, DPA *P* = 0·045 and ALA *P* = 0·051. The *P*-values for interaction suggest differences between men and women, and therefore, analyses have been stratified by sex. The number of cases per quartile analysed by sex was deemed appropriate. Nutrients were energy-adjusted using the residual method. The basic model was adjusted for age (continuous) and area (eight city-level municipalities as strata). The multivariable model additionally adjusted for covariates derived from the 5-year follow-up survey: smoking (never, past, current: 1–19, ≥ 20 cigarettes per day, missing); BMI (< 19, ≥ 19 to < 23, ≥ 23 to < 25, ≥ 25 to < 27, ≥ 27 kg/m^2^, missing); alcohol (none, consumer: 1 to < 150 grams of ethanol/week; ≥ 150 g/week, missing) and quartile of metabolic equivalent tasks per day (hours, missing). In model 3, we additionally adjusted for potential risk factors of diet, such as fruit and vegetable consumption as well as vitamin E.^(7)^ However, as the correlation coefficients between vitamin E (0·96), vegetables (0·69) and ALA is high, we added only fruit as a covariate in model 3 for ALA. Confounding factors were selected based on evidence from previous Japanese studies and the availability of data. *P* for trend was calculated by including a continuous variable from the median value for each exposure intake in the regression model. As the history of stroke is considered a mediator, it was not included as a covariate. We performed several additional analyses adjusting for a history of hypertension and diabetes mellitus, education limited to participants in cohort I with available data and limiting dementia cases to participants with a history of stroke. Instead, supplementary analyses were performed for dementia with and without a history of stroke as proxies of vascular and non-vascular dementia. We expected that Alzheimer’s would belong to the latter type. Supplementary analyses were performed in restricted periods for cohort I (2006–2009) and cohort II (2006–2012), based on the availability of the stroke incidence registry data. Details of the JPHC stroke registry have been described previously^(27)^. Briefly, medical records (including computed tomography and/or MRI) of cohort participants at each participating hospital were reviewed. Stroke incidence was confirmed as defined by the National Survey of Stroke^(28)^. Statistical analyses were performed using STATA version 17 (STATA Corporation, College Station, TX, USA).

## Results

During 410 350 person-years of follow-up with an average follow-up of 9·4 years, 5278 cases of disabling dementia were diagnosed in this Japanese cohort. Table 1 provides an overview of the characteristics of participants 43 651 (20 002 men and 23 649 women) by quartile of fish consumption. Mean age increased by a quartile of fish consumption. Fewer male participants in the highest fish consumption quartile regularly consumed alcohol, whereas a history of diabetes and stroke was highest in this group. Fewer women in the lowest fish consumption quartile regularly consumed alcohol. Other factors such as BMI and metabolic equivalent tasks only varied slightly between fish consumption groups among women (Table 1).

**Table 1.** Basic characteristics of subjects according to consumption of fish by quartile in a Japanese cohort study (*n* 43 651)

	Q1		Q2		Q3		Q4		*P* ^*^
Men (20 002)									
Number of subjects (*n*)	5001		5000		5001		5000		
	Mean	sd	Mean	sd	Mean	sd	Mean	sd	
Age at 5-year survey in years	54·99	7·25	55·38	7·27	56·42	7·42	57·93	7·75	< 0·001
BMI (kg/m^2^)	23·61	2·88	23·56	2·77	23·41	2·75	23·51	2·84	0·053
Metabolic equivalent tasks per day	32·59	6·83	32·87	6·76	32·70	6·75	32·46	6·65	0·202
	%		%		%		%		
Current smoker (%)	47·43		49·05		49·63		45·6		< 0·001
Alcohol intake, ≥ 1 g ethanol per week (%)	71·24		72·68		73·76		68·02		< 0·001
	Mean	sd	Mean	sd	Mean	sd	Mean	sd	
Fruit and vegetable consumption	334·81	289·76	387·32	231·75	412·33	252·79	474·68	367·97	< 0·001
Vitamin E	18·71	10·66	20·97	7·70	22·61	11·09	27·26	16·64	< 0·001
	%		%		%		%		
History of stroke (%)	7·90		7·42		8·66		10·14		< 0·001
Hypertension medication (%)	14·48		17·30		18·58		22·56		< 0·001
History of diabetes (%)	4·80		5·54		6·66		8·04		< 0·001
	Mean	sd	Mean	sd	Mean	sd	Mean	sd	
Fish intake	26·22	10·73	52·53	6·71	78·74	9·01	148·59	88·87	< 0·001
PUFA fish intake	13·14	7·67	25·74	9·56	38·50	14·04	71·47	57·47	< 0·001
*n*-3 PUFA intake	2·18	0·97	2·80	0·73	3·38	1·13	4·87	2·68	< 0·001
*n*-6 PUFA intake	8·61	4·45	9·24	3·04	9·64	4·89	11·09	6·94	< 0·001
EPA intake	0·16	0·09	0·30	0·08	0·43	0·11	0·78	0·09	< 0·001
DHA intake	0·30	0·14	0·52	0·12	0·73	0·16	1·29	0·82	< 0·001
DPA intake	0·05	0·02	0·09	0·02	0·12	0·03	0·21	0·13	< 0·001
ALA intake	1·64	0·89	1·84	0·66	2·00·	1·03	2·42	1·50	< 0·001
Women (23 649)									
Number of subjects (*n*)	5913		5911		5912		5912		
Age at 5-year survey in years	56·02	7·74	56·12	7·71	57·19	7·67	58·04	7·64	< 0·001
BMI (kg/m^2^)	23·52	3·26	23·42	3·05	23·32	3·06	23·47	3·18	< 0·001
Metabolic equivalent tasks per day	31·54	5·68	32·08	5·65	32·05	5·63	31·84	5·68	< 0·001
	%		%		%		%		
Current smoker (%)	6·65		5·90		5·33		5·77		0·108
Alcohol intake, ≥ 1 g ethanol per week (%)	16·08		19·45		18·8		18·18		< 0·001
	Mean	sd	Mean	sd	Mean	sd	Mean	sd	
Fruit and vegetable consumption	464·21	309·75	519·36	284·13	526·45	270·94	579·75	440·02	< 0·001
Vitamin E	21·18	10·95	23·12	8·37	23·86	7·86	28·05	17·26	< 0·001
	%		%		%		%		
History of stroke (%)	5·19		4·65		4·89		5·92		0·012
Hypertension medication (%)	17·08		18·57		20·42		21·70		0·001
History of diabetes (%)	3·08		2·88		2·88		4·01		0·001
	Mean	sd	Mean	sd	Mean	sd	Mean	sd	
Fish intake	26·82	10·95	52·79	6·50	77·42	8·03	139·76	88·89	< 0·001
PUFA fish intake	13·11	7·77	26·03	9·48	38·41	13·43	69·54	56·29	< 0·001
*n*-3 PUFA intake	2·38	0·96	3·00	0·76	3·48	0·76	4·87	2·76	< 0·001
*n*-6 PUFA intake	9·03	4·37	9·52	3·36	9·66	3·10	10·94	6·92	< 0·001
EPA intake	0·17	0·08	0·31	0·08	0·44	0·10	0·77	0·54	< 0·001
DHA intake	0·30	0·13	0·52	0·11	0·73	0·14	1·25	0·83	< 0·001
DPA intake	0·05	0·02	0·09	0·02	0·12	0·03	0·20	0·13	< 0·001
ALA intake	1·86	0·89	2·03	0·75	2·10	0·67	2·48	1·50	< 0·001

ALA, alpha-linolenic acid; DPA, docosapentaenoic acid; EPA, eicosapentaenoic acid; Q, quartile.

Food items and nutrients are reported in grams/day.

* ANOVA or *X*^2^ test.

Table 2 presents the HR, 95 % CI and *P* for trend for energy-adjusted fish intake in grams per day by quartile and the risk of disabling dementia for men and women separately. Findings are also reported separately for PUFA-rich fish, total *n*-3 PUFA and *n*-6 PUFA. Results were overall NS among men. However, we observed an association between fish and fish-related nutrients and *n*-6 PUFA and disabling dementia among women (Table 2). Compared with women in the lowest fish consumption quartile, Q1, the multivariable-adjusted HR (HR2) was significant for Q3 = 0·90 (0·81, 0·99) only. A similar pattern was observed for PUFA-rich fish with only Q2 and Q3 HR being significant in the multivariable-adjusted model (HR 0·88). Similarly, *n*-3 PUFA only Q3 was significant with a 13 % risk reduction in women. The multivariable-adjusted HR (HR2) were Q2 = 0·88 (0·79, 0·97), Q3 = 0·81 (0·74, 0·90) and Q4 = 0·90 (0·81, 0·99) with a *P* for trend = 0·024 for *n*-6 PUFA among women. In the analysis among men only, Q2 = 0·86 (0·76, 0·97) was significant. We further subdivided *n*-3 PUFA exposure into EPA, DHA, DPA and ALA in Table 3. Among women, a significant risk reduction was observed in multivariable-adjusted models: for EPA in Q2 = 0·89 (95 % CI 0·80, 0·99) and Q3 = 0·89 (0·80, 0·99), for DHA in Q3 = 0·85 (0·77, 0·95) and DPA in Q3 = 0·88 (0·80, 0·98), for ALA in Q2 = 0·90 (0·81, 0·99) and Q3 = 0·86 (0·78, 0·95), respectively, with only a significant *P* for trend of 0·043 for ALA. Among men, only Q2 showed a significant risk reduction for disabling dementia for EPA (HR 0·86, 95 % CI 0·75, 0·98), DPA (0·85, 0·75, 0·98) and ALA (0·79, 0·69, 0·89) respectively. After additionally adjusting for fruit and vegetable consumption as well as vitamin E in model 3, results largely attenuated except for *n*-6 PUFA in women. In men, Q2 remained significant for EPA (HR3 = 0·87, 0·76, 0·99) and only fruit for ALA (0·80, 0·71, 0·91), while *n*-6 PUFA Q3 = 0·81 (0·70, 0·93) and DHA in Q3 = 0·89 (0·80, 0·99) were significant among women. Results remained after additionally adjusting for history of hypertension and diabetes. In the sensitivity analysis including only participants in cohort I, adjusting for education, most significant associations attenuated except PUFA-rich fish Q2 and Q3, *n*-6 PUFA Q3 and ALA Q2 and Q3 among women and ALA Q2 among men. We performed supplementary analysis for dementia-type-specific analysis, limiting cases to participants with a history of stroke (*n* 713). Only *n*-6 PUFA Q3 remained significant and ALA *P* for trend in women.

**Table 2.** HR and 95 % CI for the association between fish, *n*-3 and *n*-6 PUFA and disabling dementia risk in a Japanese cohort study (*n* 43 651)

Food group	Participants	Median^*^	HR1^†^	95 %CI	HR2^‡^	95 %CI		HR3^§^	95 %CI	
Men (20 002)										
Total fish										
Q1	5001	28·27	1·00	Reference	1·00	Reference		1·00	Reference	
Q2	5000	52·37	0·95	0·83, 1·07	0·97	0·85, 1·10		0·99	0·87, 1·12	
Q3	5001	78·01	0·94	0·83, 1·07	0·97	0·85, 1·10		0·99	0·87, 1·13	
Q4	5000	125·28	1·00	0·89, 1·13	1·04	0·92, 1·17		1·05	0·92, 1·20	
* P* ^||^			0·702		0·385			0·324		
PUFA-rich fish										
Q1	5001	13·09	1·00	Reference	1·00	Reference		1·00	Reference	
Q2	5000	25·68	0·87	0·77, 0·99	0·90	0·80, 1·03		0·92	0·81, 1·05	
Q3	5001	37·92	0·88	0·78, 1·00	0·92	0·81, 1·04		0·94	0·82, 1·07	
Q4	5000	60·64	0·98	0·87, 1·10	1·02	0·90, 1·15		1·03	0·91, 1·17	
* P*			0·647		0·346			0·284		
Total *n*-3 PUFA										
Q1	5001	2·13	1·00	Reference	1·00	Reference		1·00	Reference	
Q2	5000	2·74	0·90	0·79, 1·02	0·94	0·83, 1·07		0·99	0·86, 1·15	
Q3	5001	3·31	0·87	0·77, 0·99	0·94	0·83, 1·06		0·97	0·83, 1·14	
Q4	5000	4·332	0·96	0·85, 1·08	1·03	0·91, 1·16		1·03	0·87, 1·23	
* P*			0·778		0·449			0·625		
Total *n*-6 PUFA										
Q1	5001	8·17	1·00	Reference	1·00	Reference		1·00	Reference	
Q2	5000	8·93	0·82	0·73, 0·93	0·86	0·76, 0·97		0·85	0·72, 1·00	
Q3	5001	9·25	0·83	0·74, 0·94	0·89	0·79, 1·01		0·82	0·67, 1·00	
Q4	5000	9·75	0·90	0·80, 1·00	0·97	0·86, 1·10		0·84	0·66, 1·06	
* P*			0·185		0·975			0·277		
Women (23 649)										
Total fish										
Q1	5913	28·95	1·00	Reference	1·00	Reference		1·00	Reference	
Q2	5912	52·7	0·90	0·81, 0·99	0·92	0·83, 1·02		0·95	0·86, 1·06	
Q3	5912	76·87	0·88	0·79, 0·97	0·90	0·81, 0·99		0·94	0·84, 1·04	
Q4	5912	118·68	0·95	0·86, 1·05	0·96	0·87, 1·06		1·00	0·91, 1·12	
* P*			0·583		0·680			0·690		
PUFA-rich fish										
Q1	5913	13·07	1·00	Reference	1·00	Reference		1·00	Reference	
Q2	5912	25·68	0·85	0·77, 0·95	0·88	0·79, 0·97		0·90	0·81, 1·00	
Q3	5912	37·92	0·87	0·78, 0·96	0·88	0·79, 0·97		0·91	0·82, 1·01	
Q4	5912	60·64	0·92	0·83, 1·01	0·92	0·83, 1·02		0·96	0·87, 1·06	
* P*			0·374		0·319			0·826		
Total *n*-3 PUFA										
Q1	5913	2·38	1·00	Reference	1·00	Reference		1·00	Reference	
Q2	5912	2·92	0·89	0·80, 0·98	0·92	0·83, 1·02		0·99	0·90, 1·11	
Q3	5912	3·43	0·85	0·77, 0·94	0·87	0·80, 0·98		0·97	0·86, 1·11	
Q4	5912	4·33	0·91	0·83, 1·00	0·93	0·85, 1·03		1·03	0·86, 1·09	
* P*			0·071		0·183			0·639		
Total *n*-6 PUFA										
Q1	5913	8·85	1·00	Reference	1·00	Reference		1·00	Reference	
Q2	5912	9·23	0·85	0·77, 0·94	0·88	0·79, 0·97		0·89	0·79, 1·00	
Q3	5912	9·38	0·78	0·71, 0·86	0·81	0·74, 0·90		0·81	0·70, 0·93	
Q4	5912	9·57	0·87	0·79, 0·97	0·90	0·81, 0·99		0·87	0·73, 1·04	
* P*			0·004		0·024			0·165		

HR, hazard ratio; Q, quartile.

* Dietary items in log energy-adjusted grams/day.

† HR1 adjusted for age and area (eight city-level municipalities as strata).

‡ HR2 multivariate hazard ratios additionally adjusted for smoking (never, past, current: 1–19, ≥ 20 cigarettes per day, missing); BMI (< 19, ≥ 19 to < 23, ≥ 23 to < 25, ≥ 25 to < 27, ≥ 27 kg/m^2^, missing); alcohol (none, past, consumer: 1 to < 150 g of ethanol/week; ≥ 150 g/week, missing) and quartile of metabolic equivalent tasks per day (hours, missing).

§ HR3 multivariate HR additionally adjusted for fruit and vegetable intake and vitamin E.

|| Median value of each quartile was included to compute the trend *P*s.

**Table 3.** Hazard ratios (HR) and 95 % CI for the association between *n*-3 PUFA EPA, DHA, DPA and ALA and disabling dementia risk in a Japanese cohort study (*n* 43 651)

Food	Participants	Median^*^	HR1^†^	95 % CI	HR2^‡^	95 % CI	HR3^§^	95 % CI
Men (20 002)								
EPA								
Q1	5001	0·17	1·00	Reference	1·00	Reference	1·00	Reference
Q2	5000	0·3	0·83	0·73, 0·95	0·86	0·75, 0·98	0·87	0·76, 0·99
Q3	5001	0·43	0·89	0·78, 1·01	0·91	0·81, 1·04	0·93	0·81, 1·07
Q4	5000	0·68	0·95	0·84, 1·07	0·98	0·87, 1·11	0·99	0·87, 1·13
* P* ^||^			0·825		0·506		0·443	
DHA								
Q1	5001	0·3	1·00	Reference	1·00	Reference	1·00	Reference
Q2	5000	0·51	0·90	0·79, 1·03	0·93	0·82, 1·06	0·95	0·83, 1·08
Q3	5001	0·73	0·94	0·83, 1·07	0·97	0·85, 1·10	1·00	0·87, 1·14
Q4	5000	1·12	1·01	0·89, 1·14	1·03	0·91, 1·17	1·05	0·92, 1·20
* P*			0·449		0·278		0·227	
DPA								
Q1	5001	0·05	1·00	Reference	1·00	Reference	1·00	Reference
Q2	5000	0·09	0·82	0·72, 0·94	0·85	0·75, 0·97	0·87	0·76, 0·99
Q3	5001	0·12	0·88	0·77, 0·99	0·90	0·79, 1·02	0·92	0·81, 1·05
Q4	5000	0·18	0·96	0·75, 1·09	1·00	0·89, 1·13	1·01	0·89, 1·16
* P*			0·615		0·320		0·267	
ALA								
Q1	5001	1·54	1·00	Reference	1·00	Reference	1·00	Reference
Q2	5000	1·77	0·75	0·66, 0·85	0·79	0·69, 0·89	0·80	0·71, 0·91
Q3	5001	1·91	0·88	0·78, 0·99	0·95	0·84, 1·07	0·97	0·86, 1·10
Q4	5000	2·13	0·89	0·79, 1·00	0·98	0·87, 1·11	1·00	0·89, 1·14
* P*			0·389		0·513		0·961	
Women (23 649)								
EPA								
Q1	5913	0·17	1·00	Reference	1·00	Reference	1·00	Reference
Q2	5912	0·3	0·88	0·79, 0·98	0·89	0·80, 0·99	0·92	0·83, 1·02
Q3	5912	0·44	0·88	0·79, 0·97	0·89	0·80, 0·99	0·93	0·83, 1·04
Q4	5912	0·67	0·94	0·84, 1·04	0·94	0·81, 1·04	0·98	0·88, 1·09
* P*			0·538		0·581		0·825	
DHA								
Q1	5913	0·3	1·00	Reference	1·00	Reference	1·00	Reference
Q2	5912	0·51	0·90	0·81, 1·00	0·92	0·83, 1·02	0·95	0·85, 1·05
Q3	5912	0·73	0·84	0·76, 0·93	0·85	0·77, 0·95	0·89	0·80, 0·99
Q4	5912	1·09	0·95	0·86, 1·05	0·96	0·86, 1·06	1·00	0·90, 1·11
* P*			0·500		0·579		0·85	
DPA								
Q1	5913	0·05	1·00	Reference	1·00	Reference	1·00	Reference
Q2	5912	0·09	0·89	0·80, 0·99	0·91	0·82, 1·01	0·93	0·84, 1·04
Q3	5912	0·12	0·87	0·78, 0·96	0·88	0·80, 0·98	0·92	0·83, 1·02
Q4	5912	0·17	0·96	0·75, 1·06	0·97	0·87, 1·07	1·01	0·91, 1·12
* P*			0·810		0·888		0·543	
ALA								
Q1	5913	1·81	1·00	Reference	1·00	Reference	1·00	Reference
Q2	5912	1·96	0·87	0·78, 0·96	0·90	0·81, 0·99	0·92	0·83, 1·01
Q3	5912	2·04	0·82	0·75, 0·91	0·86	0·78, 0·95	0·88	0·79, 0·97
Q4	5912	2·19	0·87	0·79, 0·96	0·91	0·82, 1·00	0·95	0·85, 1·04
* P*			0·004		0·043		0·192	

Abbreviations: ALA, alpha-linolenic acid; DPA, docosapentaenoic acid; HR, hazard ratio; Q, quartile.

* Dietary items in log energy-adjusted g/day.

† HR1 adjusted for age and area (eight city-level municipalities as strata).

‡ HR2 multivariate hazard ratios additionally adjusted for smoking (never, past, current: 1–19, ≥ 20 cigarettes per day, missing); BMI (< 19, ≥ 19 to < 23, ≥ 23 to < 25, ≥ 25 to < 27, ≥ 27 kg/m^2^, missing); alcohol (none, consumer: 1 to < 150 g of ethanol/week; ≥ 150 g/week, missing) and quartile of metabolic equivalent tasks per day (hours, missing).

§ HR3 multivariate hazard ratios additionally adjusted for fruit and vegetable intake and vitamin E. For ALA, we only additionally adjusted for fruit intake due to internal correlation.

|| Median value of each quartile was included to compute the trend *P*s.

## Discussion

This Japanese cohort study is one of only a few studies conducted in Asia to include many disabling dementia cases, with detailed exposure information for fish food items and related nutrients, such as PUFA intake level. Like countries with a predominantly Western diet, participants consuming more fish also had a higher traditional Japanese dietary pattern score, considered to be healthier than Western diets^(29)^. Overall, we found no association with disabling dementia. This result is in good agreement with the non-linear associations often seen in CVD^(30)^. In Japanese, the association may be undetectable because most people eat fish above the threshold.

Several mechanisms have been proposed to explain how the nutrients *n*-6 PUFA and ALA – whose benefits were suggested in women in the present study– reduce the risk of disabling dementia. Prostaglandins (PGE1) from *n*-6 PUFA decrease the inflammatory response^(31)^. ALA, an *n*-3 PUFA essential fatty acid derived mainly from plants, is a precursor which can be converted into DHA and EPA^(32–34)^ and is associated with health benefits^(19,35,36)^. Conversion may be influenced by genetic variation and sex, as well as other factors^(37,38)^. Some studies suggest that conversion may be more efficient among women^(39)^. Independently of DHA or EPA, ALA may also have a neuroprotective effect on learning^(40,41)^ and neural-related death^(42)^. The main contributors of ALA in the Japanese diet are soyabean oil (6100 mg/100 g edible portion) and vegetable oil (6800 mg/100 g edible portion)^(43)^. While individual *n*-3 associations are out of the primary scope of this study, they provide important insights into fish-related exposures. Fish consumption overall may not be associated with dementia in a population with high intake, whereas ALA, a related nutrient mainly plant-derived may reduce inflammation and protect brain cells. After adjusting for fruit intake, the inverse association between ALA and disabling dementia in women only remained for medium intake. Due to the high correlation between vegetables, vitamin E and ALA, we cannot separate the effects from these foods.

The 2017 Japanese nested case-control Circulatory Risk in Communities Study (CIRCS) also observed an inverse association between serum ALA, but not EPA or DHA, and the risk of disabling dementia^(19)^. Two cross-sectional studies in humans (Italian and Korean)^(10,44)^ and two rodent studies^(40,41)^ support these findings, while two prospective studies (USA and France) – albeit with short follow-up times and limited numbers of cases – do not^(45,46)^. Moreover, the Rotterdam study found no association while the Chicago Health and Aging Project reported only a weak inverse association between ALA and the risk of dementia in an age-adjusted but not a multivariable-adjusted model^(47,48)^. The discrepancy in study findings for ALA and dementia are not clear but might be partially due to the non-linear characteristic of the associations. As previously mentioned, subjects in the highest consumption category have many risk factors for dementia. Additionally, differences in age at food intake assessment, case number, ethnicity, follow-up duration, outcome assessment and setting may explain these differences^(49)^. If inflammation is a possible mechanism, additional dietary components may contribute to the association. Therefore, we conducted additional analyses, adjusting for fruit and vegetable consumption as well as vitamin E. HR attenuated possibly due to the adjustment for vitamin E and fruit and vegetables which may contribute to reducing the risk of disabling dementia according to a 2024 JPHC study^(7)^.

The key strength of this study is that the data is sourced from a general population with long follow-up. A second important strength is that disabling dementia cases were obtained from long-term care insurance and a universal compulsory insurance system. Third, the study includes one of the largest numbers of disabling dementia cases in the world. Fourthly, diagnoses of disabling dementia were based on evaluation by attending physicians, validated in a previous study^(50)^.

Despite these strengths, some limitations should also be considered. First, an important drawback is that we were unable to classify disabling dementia into Alzheimer’s and vascular cases. Instead, we classified cases into those with and without a history of stroke, which should correspond to vascular and non-vascular dementia^(19)^. Second, exposure and covariate data, although validated, were self-reported and thus, a degree of measurement error may have been introduced. The FFQ may not have fully captured *n*-3 PUFA levels, as it might also be sourced from metabolism. Third, we did not survey participants for prevalent dementia at baseline. Instead, we set the baseline at least nine years before the beginning of dementia ascertainment. Misclassification using the dementia rating scale could not be completely eliminated. Misclassification may have been affected by false-negative diagnoses, underestimating the actual number of cases. Reverse causation can also not be completely excluded. Lastly, other dementia cases may have been missed using the eligibility for insurance support approach, leading to an underestimation of the true number of cases.

In conclusion, we found that overall fish consumption, as well as PUFA intake, was not associated with the risk of disabling dementia. Our findings suggest that *n*-6 PUFA and ALA may be associated with disabling dementia in Japanese women, bearing in mind the high distribution of fish consumption in Japan must be interpreted with care and warrants confirmation in future studies.
